# Frequency of Fractures Identified on Post-Reduction Radiographs After Shoulder Dislocation

**DOI:** 10.5811/westjem.2015.11.28855

**Published:** 2016-01-21

**Authors:** Michael Gottlieb, Damali Nakitende, Laurie Krass, Anupam Basu, Errick Christian, John Bailitz

**Affiliations:** Cook County Hospital, Department of Emergency Medicine, Chicago, Illinois

## Abstract

**Introduction:**

Most emergency physicians routinely obtain shoulder radiographs before and after shoulder dislocations. However, currently there is limited literature demonstrating how frequently new fractures are identified on post-reduction radiographs. The primary objective of this study was to determine the frequency of new, clinically significant fractures identified on post-reduction radiographs with a secondary outcome assessing total new fractures identified.

**Methods:**

We conducted a retrospective chart review using appropriate International Classification of Diseases, 9^th^ Revision (ICD-9) codes to identify all potential shoulder dislocations that were reduced in a single, urban, academic emergency department (ED) over a five-year period. We excluded cases that required operative reduction, had associated proximal humeral head or shaft fractures, or were missing one or more shoulder radiograph reports. All charts were abstracted separately by two study investigators with disagreements settled by consensus among three investigators. Images from indeterminate cases were reviewed by a radiology attending physician with musculoskeletal expertise. The primary outcome was the percentage of new, clinically significant fractures defined as those altering acute ED management. Secondary outcomes included percentage of new fractures of any type.

**Results:**

We identified 185 total patients meeting our study criteria. There were no new, clinically significant fractures on post-reduction radiographs. There were 13 (7.0%; 95% CI [3.3%–10.7%]) total new fractures identified, all of which were without clinical significance for acute ED management.

**Conclusion:**

Post-reduction radiographs do not appear to identify any new, clinically significant fractures. Practitioners should re-consider the use of routine post-reduction radiographs in the ED setting for shoulder dislocations.

## INTRODUCTION

Shoulder dislocations are a common emergency department (ED) presentation, affecting 1.7% of the population.^1^,^2^ Dislocations occur due to a variety of both traumatic and atraumatic causes ranging from falling onto an outstretched arm to reaching over to pick up a telephone. Shoulder dislocations often recur, especially in young adults, so it is not unusual for a patient to present repeatedly to the ED for this problem.^1^

Most emergency physicians (EP) routinely obtain shoulder radiographs before and after shoulder dislocations to assess for persistent dislocations and fractures. Prior studies have demonstrated that EPs are able to detect reductions clinically with excellent accuracy.^3^–^5^ Additionally, the identification of new fractures on post-reduction imaging has been suggested to be low in prior studies, though most are limited by small sample sizes.^4^–^8^ As a result, it has been suggested that the post-reduction radiograph may not be necessary in the ED environment.^8^

Decreasing the number of radiographs obtained would save time, reduce radiation exposure, and lower health care costs. With increased focus on cost containment, throughput times, and radiation exposure, there is a need to re-evaluate our current approach to many traditional procedures in the ED. The objective of this study was to determine the frequency of new, clinically significant fractures identified on post-reduction radiographs.

## METHODS

This study was conducted in an urban, tertiary care, ED associated with an emergency medicine residency program, with an annual ED census of 120,000 patients. We conducted a retrospective chart review of all cases of shoulder dislocation seen in the ED between November 2010 (the first available electronic medical record) and March 2015. A search of all International Classification of Diseases, 9th Revision (ICD-9) discharge codes relevant to shoulder dislocation was performed to generate the initial patient list. The inclusion criteria required an evaluation in the ED for shoulder dislocation, complete medical record, and both pre- and post-reduction images. We excluded cases with missing images or those requiring operative reduction. Patients with pre-reduction fractures not described above (i.e. Hill-Sachs, Bankart, or greater tuberosity fracture) were included in this study.

Each chart was reviewed independently by two study investigators and entered into a data collection form. All disagreements were settled by consensus among three investigators. Age, sex, past medical history, reduction technique, pre-reduction radiograph findings, and post-reduction radiograph findings were all extracted and subsequently entered into the study database. Any new fractures or indeterminate cases were reviewed by an attending radiologist with musculoskeletal expertise who was blinded to the case data.

The primary outcome was the percentage of new, clinically significant fractures on post-reduction radiographs. Clinical significance was defined as a new fracture not classified as a Hill-Sachs, Bankart, or greater tuberosity fracture. Clinically significant fractures included, but were not limited to, humeral neck and shaft fractures. The decision to exclude Hill-Sachs, Bankart, and greater tuberosity fractures from the primary outcome was based upon prior evidence suggesting that these fracture types are well known to be caused by the initial dislocation mechanism and may be present in as many as two-thirds of shoulder dislocations, but are often only identified on specific orthopedic views and advanced imaging not typically performed in the ED.^9^,^10^ This definition was in conjunction with prior studies that had also excluded these fracture types from the “clinically significant” category.^3^ Moreover, these fracture types rarely affect acute ED patient management. Although identification of these fractures may increase the risk of recurrent dislocation due to joint instability, all patients were given an urgent follow-up appointment in orthopedic clinic, so it was not anticipated to alter the acute treatment or follow-up plan. The secondary outcome assessed the percentage of newly identified fractures of any type.

We calculated a sample size of 180 subjects based upon a 90% power with a two-tailed alpha=0.05 to detect a maximum new fracture rate of 3%. We performed all of the statistical analyses included in this study using Statistical Package for the Social Sciences (Version 21.0. Armonk, NY). Descriptive statistics, including population estimates at a 95% level of confidence were generated for percentage of new clinically significant fractures and percentage of total new fractures.

This study was conducted with adherence to the Statement for Reporting Studies of Diagnostic Accuracy (STARD) criteria.^11^ The local institutional review board approved this study.

## RESULTS

**Figure 1:**
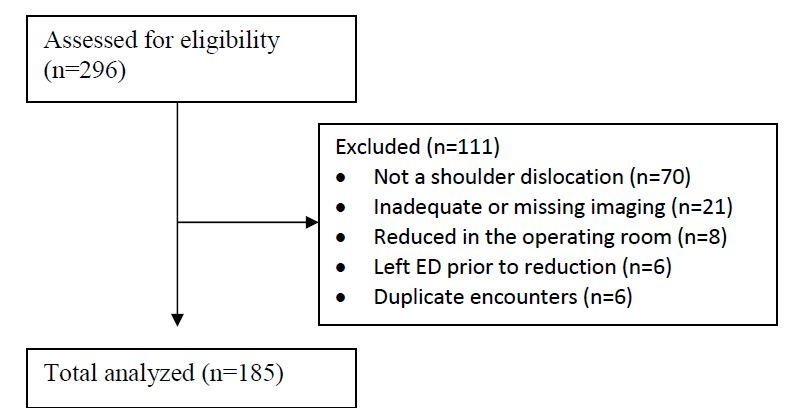
CONSORT flow diagram.

During the study period, we identified 296 patients with an ICD-9 code suggesting shoulder dislocation. Of these cases, full chart review identified 185 patients meeting the study criteria. We excluded 111 patients after initial chart review for the following reasons: 70 patients had isolated acromioclavicular joint separation or an already reduced shoulder dislocation prior to arrival; 21 patients had missing or inadequate imaging; eight patients were reduced in the operating room; six left the ED prior to having their shoulder reduced; and six records were duplicates of the same patient encounter (Figure 1).

Of the remaining 185 patients, the average age was 39 years (range: 16 to 85 years) and 80% were male. Ninety patients (48.6%) had a history of prior dislocation of the same shoulder joint. One hundred thirty-five patients (73.0%) had the reduction technique(s) described. Of these patients, 74 (54.8%) were reduced with the Kocher technique, 53 (39.2%) were reduced with traction/counter-traction, 24 (17.8%) were reduced with scapular manipulation, 14 (10.4%) were reduced with the FARES technique, eight (5.9%) were reduced with the Milch technique, five (3.7%) were reduced with the Cunningham technique, and three (2.2%) were reduced with the Stimson technique. Thirty patients (22.2%) were reduced using multiple of the aforementioned techniques.

There were no new, clinically significant fractures. There were 13 (7.0%; 95% CI [3.3%–10.7%]) total new fractures identified. Of these new fractures, 12 (6.5%) were Hill-Sachs deformities and four (2.2%) were Bankart fractures. All patients with fractures identified on post-reduction radiograph received urgent orthopedic surgery follow up and none underwent surgical intervention.

## DISCUSSION

Many physicians routinely obtain shoulder radiographs before and after reduction of shoulder dislocations. However, there is questionable yield with such practice. Given increasing pressure to reduce costs, radiation exposure, and turnaround times, this is an area that may significantly benefit from reduced imaging.

Prior studies have assessed similar aspects in an attempt to reduce the number of pre- and post-reduction radiographs obtained. The first publication was a retrospective analysis of 69 total dislocations identifying no clinically significant post-reduction fractures.^6^ Hendey and Kinlaw^3^ performed a subsequent retrospective analysis in 1996 demonstrating no clinically significant new fractures among 175 patients, while both Shuster^4^ and Hendey^5^ prospectively assessed this with smaller groups in 1999 demonstrating no clinically significant fractures in two separate studies of 45 and 98 patients, respectively. Finally, Hendey^7^ and Kahn^8^ both noted similar results in 2006 as secondary outcomes in two separate studies of 30 and 40 patients, respectively.

Our study was the largest analysis to date, consisting of 185 patients from a different patient population than most prior studies. Our patient population was urban, predominately uninsured, and of an older age group than prior studies. Additionally, we assessed this with more recent radiographic technology, which may allow for improved sensitivity compared with that of 20 years prior. We identified no new clinically significant fractures in our patient population, further strengthening existing data identifying the low utility of post-reduction radiographs for shoulder dislocations. Although there were 13 new fractures identified on the post-reduction radiograph, the majority were Hill-Sachs and Bankart fractures, which are well known to be caused by the initial dislocation and often not visible on the initial pre-reduction films.^9^,^10^ Identification of these fractures may increase the risk of recurrent dislocation due to joint instability. However, as all patients were given an urgent follow-up appointment in orthopedic clinic, it did not alter the acute ED treatment or follow up. Additionally, there is no set cutoff with regard to Hill-Sachs or Bankart deformities that triggers operative management, and most operative decisions are made in conjunction with the patient age, physical examination, and response to conservative treatment.^9^ Screening for Hill-Sachs or Bankart lesions post-reduction may be performed as an outpatient to improve ED flow.

Some physicians may not feel comfortable confirming shoulder reduction on physical examination alone. However, prior research has demonstrated that physical examination is reliable for confirming reduction.^3^–^5^ Moreover, a recent study of 73 patients demonstrated that ultrasound could reliably confirm both dislocation and reduction with 100% accuracy.^12^ Nonetheless, in cases where the physician is unsure of the reduction, post-reduction radiographs should be obtained.

## LIMITATIONS

One limitation of this study was the use of a retrospective design. Consequently, the decision to obtain imaging was dependent upon the clinical judgment of the involved providers, which may have led to a selection bias. However, only 21 patients (7.1%) identified by the study protocol did not have adequate pre-reduction and post-reduction imaging. Additionally, by using a chart review technique and searching by ICD-9 codes, it is possible that some cases may have been missed. However, given the breadth of ICD-9 codes used, it is unlikely to have been a significant proportion of cases and there is no reason to suggest that the missed patients would be substantially different than the included cases. Further, reduction techniques were not documented for some patients. However, as the primary outcome was the number of clinically significant fractures, this information was not critical to the study. Finally, many pre-reduction radiographs were missing either a lateral or scapular Y-view, which may have caused the initial fracture to be missed. If this occurred, it would have decreased the rate of new fractures, which would serve to strengthen our current conclusion.

## CONCLUSION

Post-reduction radiographs do not appear to identify any new clinically significant fractures. Practitioners should re-consider the use of routine post-reduction radiographs in the ED setting for shoulder dislocations.
